# Identification of a Subpopulation of Marrow MSC-Derived Medullary Adipocytes That Express Osteoclast-Regulating Molecules: Marrow Adipocytes Express Osteoclast Mediators

**DOI:** 10.1371/journal.pone.0108920

**Published:** 2014-10-10

**Authors:** Vance Holt, Arnold I. Caplan, Stephen E. Haynesworth

**Affiliations:** Skeletal Research Center, Department of Biology, College of Arts and Sciences, Case Western Reserve University, Cleveland, Ohio, United States of America; French Blood Institute, France

## Abstract

Increased marrow medullary adipogenesis and an associated decrease in bone mineral density, usually observed in elderly individuals, is a common characteristic in senile osteoporosis. In this study we investigated whether cells of the medullary adipocyte lineage have the potential to directly support the formation of osteoclasts, whose activity in bone leads to bone degradation. An in vitro mesenchymal stem cell (MSC)-derived medullary adipocyte lineage culture model was used to study the expression of the important osteoclast mediators RANKL, M-CSF, SDF-1, and OPG. We further assessed whether adipocytes at a specific developmental stage were capable of supporting osteoclast-like cell formation in culture. In vitro MSC-derived medullary adipocytes showed an mRNA and protein expression profile of M-CSF, RANKL, and OPG that was dependent on its developmental/metabolic stage. Furthermore, RANKL expression was observed in MSC-derived adipocytes that were at a distinct lineage stage and these cells were also capable of supporting osteoclast-like cell formation in co-cultures with peripheral blood mononuclear cells. These results suggest a connection between medullary adipocytes and osteoclast formation in vivo and may have major significance in regards to the mechanisms of decreased bone density in senile osteoporosis.

## Introduction

Bone homeostasis entails the balanced process of the generation (formation) and breakdown (resorption) of bone. This process of bone formation and resorption, or bone remodeling, is controlled by a coupling behavior between osteoblasts and osteoclasts [1]. The interaction between the osteoblast and osteoclast within remodeling marrow compartments is a developmentally regulated interaction in which each cell can induce or inhibit the developmental progression of the other cell through the direct and indirect actions of developmentally regulated factors [2]–[5]. One central factor in the coupling dynamic is receptor activator of NfκB signaling ligand (RANKL), which is expressed by early osteoblasts [6]–[8]. RANKL signals through its receptor RANK, expressed by osteoclast progenitors, which leads to the formation and maturation of osteoclasts. In bone marrow, RANKL is counteracted by its inhibitor, a soluble decoy receptor of RANKL, osteoprotegerin (OPG), which is expressed by mature osteoblasts [3].

Adipose tissue is an essential metabolic organ that has important regulatory functions in insulin sensitivity, lipid metabolism, and energy homeostasis [9], [10]. Recently, adipose tissue has been identified as having regulatory potential through its expression and secretion of cytokines and adipokines. Indeed, adipose tissue has been associated with many different physiological processes such as appetite control, inflammatory responses, and angiogenesis [11]. Dysregulation of normal adipose tissue homeostasis in various depots has been associated with physiological maladies such as hypertension, osteoporosis, diabetes, and various inflammatory diseases [12].

Medullary adipocytes are present post-natally and steadily increase in number with age [13]–[15]. In late adulthood, the majority of bone marrow volume consists of medullary adipose tissue. Interestingly, a correlation has been observed between increased bone marrow adiposity and age-associated decrease in bone mineral density [16]–[18]. This correlation between increased adiposity of the bone marrow and decreased bone density has been observed in mouse, both in vitro and in vivo [19]–[25], leading to the speculation of a possible link between adipocytes and bone remodeling via osteoclast activity [20]–[22], [26], [27].

The medullary adipocyte and osteoblast share a common progenitor cell in the mesenchymal stem cell (MSC) and have been shown to co-localize within similar regions inside marrow compartments [28]–[31]. Studies from mouse in vitro co-cultures have shown that stromal pre-adipocytes are capable of supporting osteoclast-like cell formation from osteoclast precursors [19], [24]. Recent studies have been reported in which marrow stromal RANKL expression is increased in corn oil-fed mice whose bone marrow has increased adiposity [21]. In humans, a recent study showed that primary medullary adipocytes isolated from the iliac crest marrow expressed the important osteoclast regulatory molecules OPG, M-CSF, and RANKL [25], [32]. Collectively, these data suggest a potential for medullary adipocytes to support osteoclast formation through the expression of RANKL and other osteoclastogenic mediators. However, stringent characterization of the developmental stage of the medullary adipocyte that can express these osteoclast mediators and, in turn, affect osteoclast formation has not been performed.

Characterization of bone marrow medullary adipose tissue and its role in bone metabolism is still in its early stages [23], [33], [34], and the role of adipose tissue within the marrow microenvironment remains unclear [23], [34]–[37]. Our lab was among the first to characterize the process of differentiation of MSCs into adipogenic cells and report that MSC-derived adipocytes and primary isolated medullary adipocytes share a very similar phenotypic expression profile [28]. Similar results have been reported by Qian et al. [38].

In this study, we used our well characterized MSC-derived adipocyte culture system to address the potential role of medullary adipose tissue in the regulation of bone remodeling. We hypothesized that cells of the medullary adipocyte lineage can regulate osteoclastogenesis in a developmentally regulated manner through the expression of osteoclastogenesis mediators such as RANKL, OPG, M-CSF, and SDF-1α. This study provides a novel insight into the relationship between adipocytes and osteoclasts which may give implications into further elucidating the probable role of adipocytes in age-related bone loss in senile osteoporosis.

## Materials and Methods

### Reagents

Recombinant RANKL and OPG protein was purchased from Peprotech (Rocky Hill, NJ), and DNase was purchased from Qiagen (Valencia, CA). Antibodies used were: Rabbit anti-RANKL (ab65024) and mouse anti-RUNX2 (ab76956, Abcam, Cambridge, MA), goat anti-C/EBPα antibody (sc-9314, Santa Cruz Biotechnology, Santa Cruz, CA), and mouse anti-PPARγ1/2 antibodies (ab45278, Abcam, Cambridge, MA). ELISA kits were used to detect M-CSF, SDF-1 (R&D Systems, Minneapolis, MN), and OPG (RayBiotech, Norcross, GA).

### Mesenchymal Stem Cell isolation and Culture

Iliac crest bone marrow aspirate was obtained by routine aspiration from normal human donors who gave informed written consent under an IRB-approved protocol from University Hospitals, Cleveland, OH. MSCs were isolated on the day of harvest from 10 or 20 mL marrow samples according to methods reported previously [39]. In brief, 10 mL of diluted bone marrow aspirate were centrifuged at 550 g, and the supernatant was discarded. The remaining cell suspension was loaded onto a Percoll gradient diluted to a concentration of 1.073 g/mL with Hank's balanced salt solution. After centrifugation at 900 g, mononuclear cells were removed from the interface and washed with Tyrode's salt solution for a final centrifugation at 900 g. The pelleted cells were plated for expansion in growth medium consisting of low glucose DMEM (5 mM glucose) supplemented with 10% fetal bovine serum (FBS) of selected lots [40]. These primary cells were plated at a concentration of 1.3×10^5^–2.0×10^5^ cells per cm^2^, and the medium was changed twice weekly. Just prior to their reaching confluence, MSCs were passaged at a ratio of 1∶3. Cells were incubated at 37°C in a humidified atmosphere containing 5% CO_2_. Medium changes and sub culturing procedures, as described [28], were followed for first and second passages. By second passage, contaminating hematopoietic cells were sufficiently diluted out, and cells were harvested and plated for experimentation during third passage.

### MSC-derived medullary adipocytes

MSC-derived adipocytes were isolated and sub-cultured as described above. For adipogenic induction of human MSCs, methods were used as previously described [28]. Briefly, MSCs at second or third passage were induced to form adipocytes using adipogenic induction medium containing 1 µM dexamethasone, 100 µM indomethacin, 0.5 mM 3-isobutyl-1-methylxanthine (IBMX), 10 µg/ml insulin, and 10% FBS in DMEM-high glucose for up to 12 days as determined by peak adipogenic expression and RANKL expression. At that point, the medium was changed to adipocyte maintenance medium composed of high glucose DMEM with 10 µg/ml insulin and 10% FBS to promote adipocyte maturity [28]. Cultures were analyzed prior to adipocyte induction on day 0 and at the specific time-points throughout a 25-day time-course.

### MSC-derived adipocyte culture enrichment and adipocyte ceiling culture

Lipid laden and non-lipid-laden (adipofibroblasts) cell populations were enriched by a modified method that has been previously described [28], [41], [42]. MSC-derived adipocyte cultures were trypsinized on day 12 and resuspended in adipocyte maintenance medium. For separation of cell populations, cells were centrifuged at ∼1000 rpm for 5 minutes after which the supernatant containing the lipid-laden fraction was removed and placed in a separate tube for high speed centrifugation (∼2000 rpm). The pelleted cells containing the adipofibroblasts were then resuspended in adipocyte maintenance medium and mixed vigorously, and a low speed centrifugation step was done at ∼300 rpm for 5–10 minutes. The supernatant was removed, medium replenished and the low speed centrifugation steps were repeated. The resulting adipofibroblast cells were plated for subsequent analysis, while the lipid-laden cells were placed in flasks for ceiling cultures for later analysis. The adipofibroblasts were cultured for up to 2 days before analysis.

Ceiling cultures were used to plate the enriched lipid-laden cells from MSC-derived adipocyte cultures. Isolated cells were placed in 50-mL conical tubes and suspended in adipocyte maintenance medium. The isolated cells were then placed into small T-12.5 flasks, which were filled to the top with additional medium. The flasks were then inverted and placed in 37°C incubators overnight. After overnight culturing, the medium was removed from the flasks and cells were re-fed. The flasks were then placed right side up for additional culturing time and analysis.

### Oil Red O staining

Cells were fixed with 3% paraformaldehyde for 20 min at 4°C. Cells were then rinsed and washed briefly in 60% isopropanol. Cultures were stained with 2.1 mg/ml oil red O solution for 15 minutes to stain lipid droplets/vacuoles. Cells were rinsed and destained with 100% isopropanol, and total stain was measured with a spectrophotometer at a wavelengths of 500 nm or 530 nm. To quantify staining on a per cell basis, cells were counterstained with a nuclear dye (hematoxylin or Hoechst 3342 fluorescent dye) and manually counted from random fields and averaged by total area.

### RNA isolation and quantitative PCR

DNase treated RNA was isolated from MSC-derived adipocytes, enriched adipofibroblasts and lipid laden adipocyte cultures at specific time-points using RNeasy Mini Kit according to the manufacturer's instructions (Qiagen). Cells were lysed on plate in GITC-containing buffer (Buffer RLT). Reverse Transcription was performed immediately after RNA isolation using Transcriptor First Strand cDNA synthesis kit using oligo-dT primers (Roche, Branchburg, NJ). Expression levels of the beta-actin gene were used to normalize mRNA expression for real-time PCR. Reactions were performed using the following conditions: 95°C for 10 min and cycles of 95°C for 15 sec. 55–60°C for 30 sec and 72°C for 30 seconds for 37 cycles. The primers used for each gene is listed in Table S1.

### Immunofluorescence

Cells were fixed in 3% paraformaldehyde in Tyrode's salt solution at 4°C for 20 minutes and rinsed with wash buffer (0.1% BSA in Tyrode's salt solution). Cells were then permeabilized with 0.01% digitonin in Tyrode's salt solution for 20 minutes and rinsed in wash buffer. Blocking of non- specific antibody interaction was carried out using a 10% serum solution based on secondary antibody species. Blocking was carried out for 30–50 minutes. Anti-RANKL primary antibody in 10% goat serum was added for 1–2 hours at room temperature. For co-staining, RANKL primary antibody was added in a blocking solution with mouse derived antibodies for either Runx2, PPARγ1/2, SH2 antibody [43], [44], anti-CD90 antibody (gift from Michael Sorrell), or goat anti-C/EBPα. After washing, cells were incubated for 2 hours at room temperature with fluorophore-conjugated secondary antibodies. Cell were washed, and counterstained with 3 µg/mL Hoechst 33342 (Sigma) prior to imaging. Quantitative analysis was done by counting RANKL positive cells in 4–5 random fields (n = 3–4 donors). All images were processed using Image J for quantitative analysis and Adobe Lightroom for contrasting.

### PBMNC isolation and osteoclast co-cultures

Peripheral blood from separate donors was diluted 1∶1 with PBS and layered over a Ficoll-Paque solution at room temperature. The Ficoll-gradient solution was then centrifuged at 1500 rpm for 30 minutes The peripheral blood mononuclear cells (PBMNC) were collected from the Ficoll-Paque/plasma interface, washed with PBS and used immediately for analysis. To generate osteoclasts, PBMNC were plated at either 0.5 or 0.8×10^6^ cells/cm^2^ on 48-well or 24-well plates in RPMI medium supplemented with 10% FBS. M-CSF (50 ng/ml) and RANKL (60 ng/ml) were added. Osteoclast cultures were fed every 3 days up to 14 days. At specified times (days 0, 3, 9, and 14) cells were fixed and stained using TRAP staining kit as described by the manufacturer (Sigma-Aldrich) and counterstained with hematoxylin. For co-culture experiments, PBMNC were placed on confluent layers of either adipofibroblasts or dermal fibroblasts and cultured for up to 21 days in RPMI: DMEM (1/1) medium supplemented with 10% FBS.

## Results

### RANKL, OPG, and M-CSF mRNA expression profile in adipocyte lineage progression

**Figure 1:**
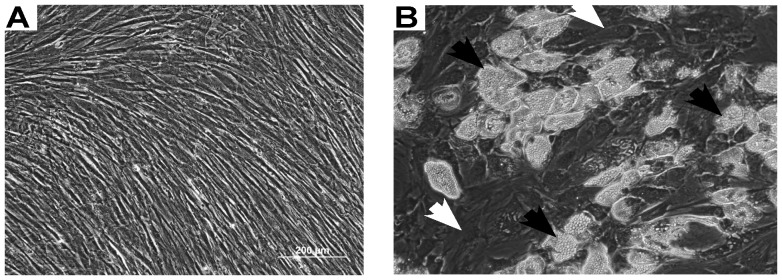
Human bone marrow-derived cells and their differentiation to the adipocyte lineage. A) Phase contrast image of bone marrow-derived mesenchymal stem cells grown for 12 days in growth medium. B) Phase contrast images of parallel cultures grown in adipogenic medium for 12 days. MSC-derived adipocyte cultures show a heterogenous mixture of cell morphologies including lipid-laden cells (*black arrows*) and non-lipid laden cells (*white arrows*).

**Figure 2:**
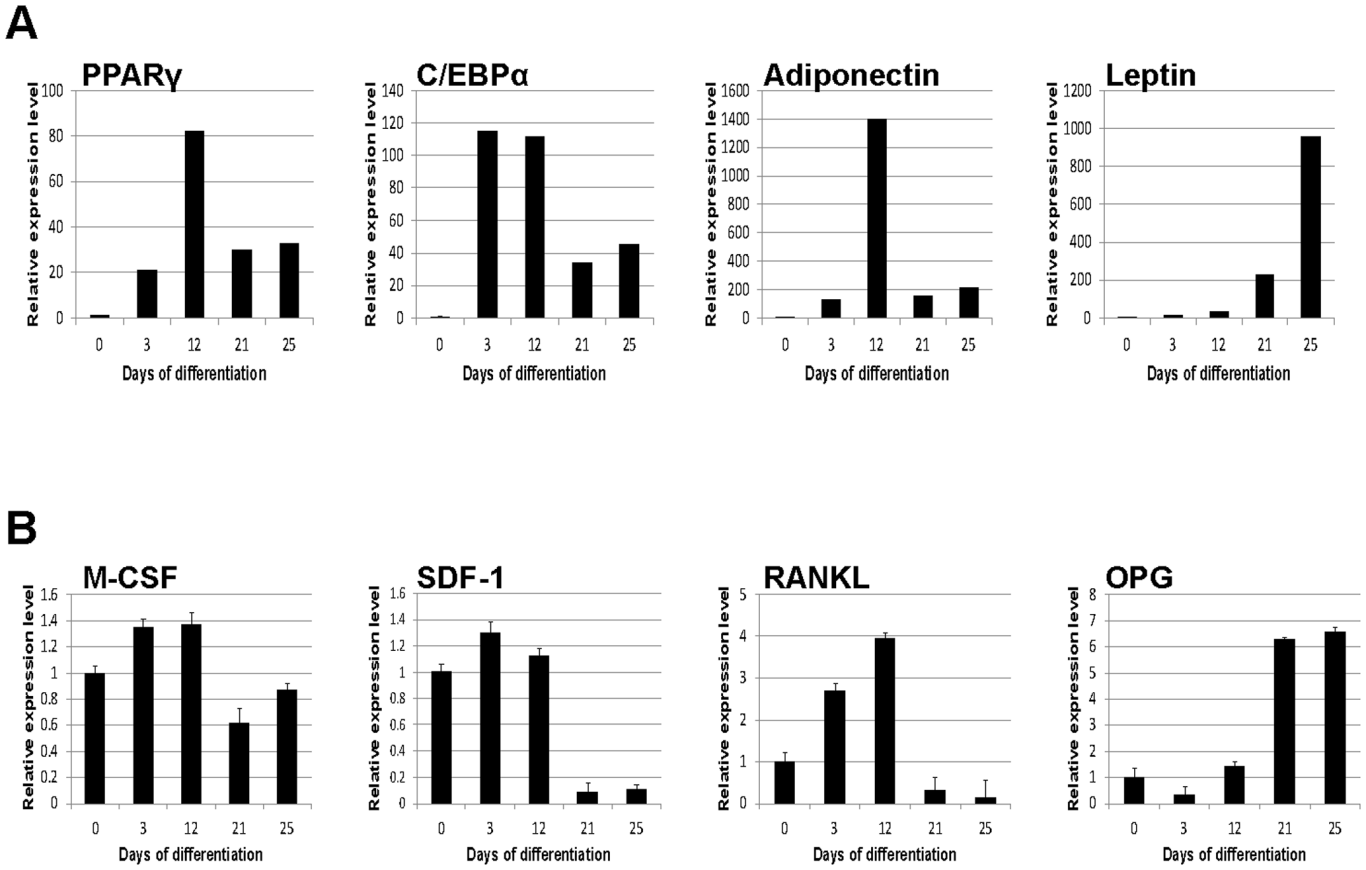
Adipogenic lineage markers and osteoclast mediator expression profile in MSC-derived adipocytes. Total RNA from MSC-derived medullary adipocytes was isolated during the 25-day differentiation process and analyzed for (**A**) known adipogenic lineage markers PPARγ2 C/EBPα, adiponectin, and leptin. (**B**) The expression profiles of regulators of osteoclast development were mapped using the same analysis to quantify M-CSF, SDF-1, RANKL, and OPG. Measurements of transcript levels were performed using quantitative RT-PCR and graphed relative to levels of transcripts in MSCs at day 0. Error bars indicate variation between samples and is representative of all donors tested. *n* = *3–4 donors*.

Mesenchymal stem cells (MSCs) progressively differentiate into mature medullary adipocytes in vitro during a 24-day time course (**Figure 1**). In order to map the progression of adipogenic differentiation, cell layers for mRNA isolation and conditioned medium were collected at specified times during the 24-day time course. Gene expression of the adipogenic developmental stage markers was measured using quantitative RT-PCR. PPARγ2, C/EBPα, adiponectin, and leptin each generated distinct patterns of mRNA expression during adipocyte differentiation (**Figure 2A**). PPARγ2 and C/EBPα expression was induced early (day 3) and was high in early stages of adipogenic differentiation (up to day 12), whereas significant adiponectin expression was observed at the mid-stage (day 12) of adipogenic development [28]. Leptin expression in adipocytes has been associated with adipocyte maturity [28], [45], and indeed there were high levels of leptin at 21–25 days of differentiation, times when PPARγ2, C/EBPα, and adiponectin levels were decreased.

In order to determine whether the genes for the osteoclast mediators M-CSF, RANKL, and OPG are expressed in a developmentally regulated manner, mRNA for these genes was measured using quantitative RT-PCR (**Fig. 2B**). The MSC phenotype changes significantly after extended time in culture at a high-confluence; including changes in RUNX2, collagen I, osteocalcin, and RANKL expression (Fig. S1A-D). Thus, mRNA expression of the MSC-derived adipocyte was compared to day 0 MSC cultures which is believed to more closely represent an uncommitted MSC. M-CSF mRNA expression was increased initially up to day 12 of differentiation n, and then dropped during the mature adipogenic stages (days 21 and 25). SDF-1 mRNA levels were unchanged in early stages, but significantly decreased at days 21 and 25 of adipogenic development. RANKL mRNA levels were significantly induced in the adipocyte cultures compared to MSC controls at days 3 and 12. However, during the timecourse of adipogenic induction, RANKL expression increased up to day 12, and then dramatically declined during the late adipogenesis (days 21 and 25). Conversely, OPG mRNA expression was low during the early phases and dramatically increased after day 12 of differentiation. These results indicate that important osteoclastogenic mediators have a distinct temporal expression profiles in adipocytes that correlate with the MSC-derived adipocyte lineage stage.

### RANKL SDF-1, M-CSF, and OPG protein expression by MSC-derived medullary adipocytes

**Figure 3:**
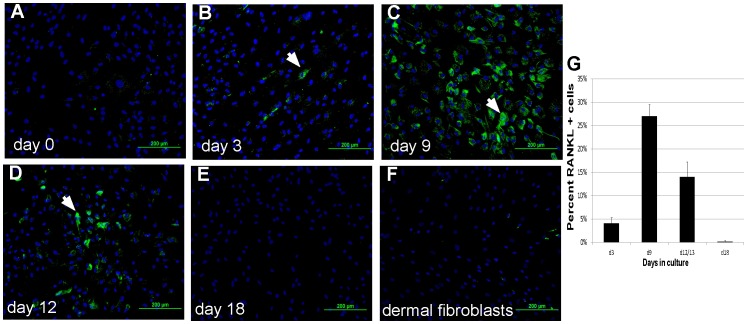
RANKL protein is regulated during adipocyte differentiation. MSC cultures were induced to form MSC-derived adipocytes, and RANKL protein was detected using immunofluorescence. Cells were counterstained with Hoechst33342, and RANKL protein levels were quantified per cell number by manual counting. Analysis was done on (**A**) day 0, (**B**) day 3, (**C**) day 9, (**D**) day 12 and (**E**) day 18. (**F**) Dermal fibroblasts in culture between 3–12 days were used as a control. There is an increase in both the number and intensity (*white arrows*) of RANKL positive cells between days 3 and 12 after differentiation. Scale bar in panel A = 200 µm for all panels. (**G**) To quantify changes in RANKL protein levels cells, the percentage of stained cells was counted at different times in culture. *n* = *3–4 donors*.

**Figure 4:**
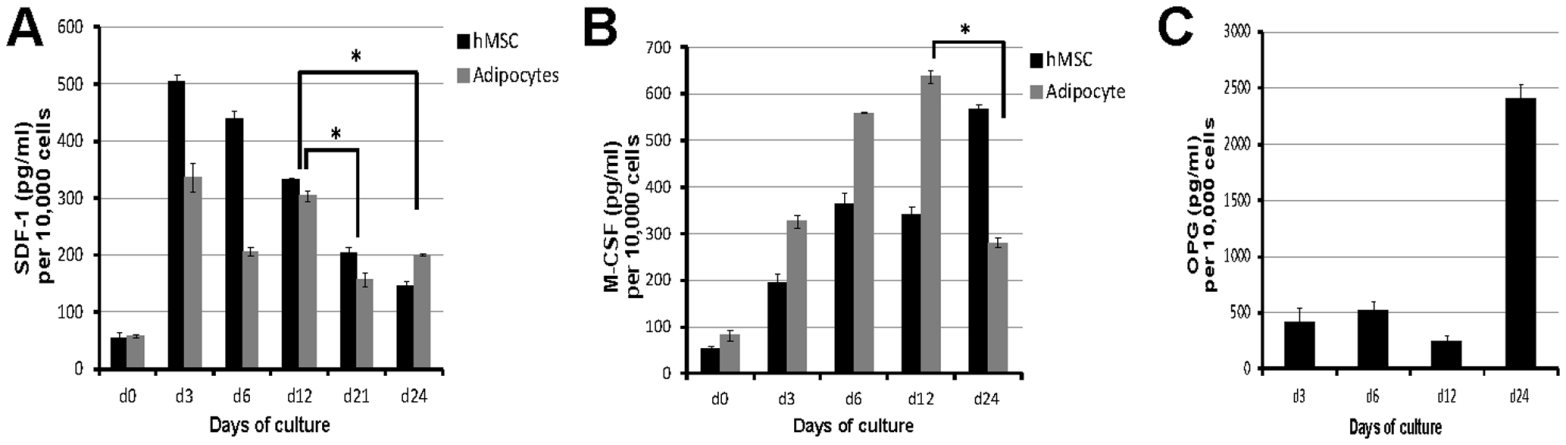
Production of osteoclast-regulating molecules during adipogenesis 3-day conditioned medium from high density untreated MSC in control medium and MSC-derived adipocyte cultures were collected at days 3, 6, 12, and 24. An ELISA was performed to quantify (A) SDF-1, (B) M-CSF, and (C) OPG. Representative graphs from each experiment show that protein levels of SDF-1 and M-CSF within conditioned medium from MSC-derived adipocytes is higher when compared to MSCs during the early to mid stages of culture while OPG levels are increased only during the late/mature phase of MSC-derived adipocyte cultures. * indicates a p-value <0.05.

In order to confirm the mRNA expression profiles, protein expression was analyzed in MSC-derived adipocytes at the same culture-defined adipocyte lineage stages. Cell surface RANKL was analyzed using immunofluorescence (**Fig. 3**). RANKL was increased at day 3 (**Fig. 3B**), day 9 (**Fig. 3C**), and day 12 (**Fig. 3D**) compared to MSC (**Fig. 3A**) and dermal fibroblast controls (**Fig. 3F**). Quantification of the percentage of RANKL-positive cells (**Fig. 3G**) shows that RANKL protein has a distinct developmentally regulated profile similar to its pattern of mRNA expression. Secreted SDF-1, M-CSF, and OPG was measured in 3-day conditioned medium using ELISA for each protein (**Fig. 4**). The levels of these proteins were normalized to cell number, and compared between high density untreated MSC (hMSC) and cells under adipocyte differentiating conditions (adipocyte). SDF-1, M-CSF, and OPG protein were each detected in conditioned medium at varying levels based on adipogenic stages and generated profiles similar to the mRNA expression patterns of each factor. Notably, SDF-1 in medullary adipocytes never exceeded levels in MSC, which are known to produce significant levels of SDF-1. M-CSF protein was significantly elevated in the first 12 days of adipocyte differentiation, but was low in mature adipocyte conditioned medium at day 24 (**Fig. 4B**). OPG protein levels were low in early MSC-derived adipocyte cultures (days 3–12) but was markedly increased during the late stages (day 24) (**Fig. 4C**).

### RANKL, SDF-1, and M-CSF expression in distinct adipocyte populations

**Figure 5:**
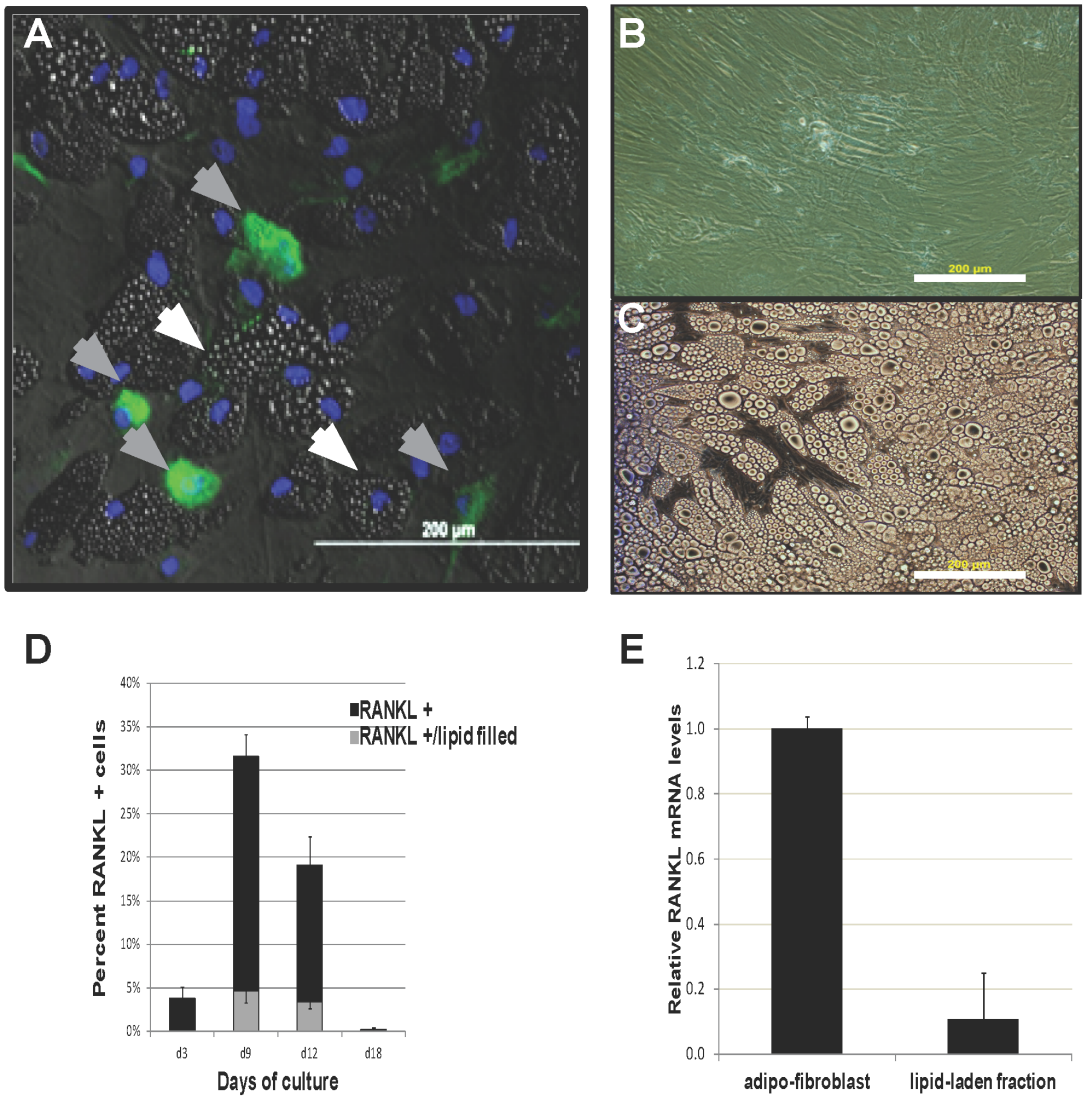
RANKL is present in a sub-population of adipocytes. (**A**) Immunostaining was performed in MSC-derived adipocyes at day 12 of differentiation (green). Two sub-populations were identified based on the presence (white arrows) or absence of lipid vacuoles (gray arrows). Adipocytes that show positive RANKL staining primarily were non-lipid containing cells. Cells were further sub-fractionated into (**B**) non-lipid-laden and (**C**) lipid-laden MSC-derived adipocyte populations. Scale bar in C = 200 µm for panels B-C. (**D**) RANKL protein was quantified over time in both sub-populations of cells, and confirmed that lipid-laden adipocytes represented a very low percentage of the total RANKL-positive cells. (**E**) This observation was confirmed through quantitative RT-PCR analysis to detect RANKL transcript. Lipid-laden populations (lipid fraction) showed an approximately 10-fold lower level of RANKL mRNA compared to the non-lipid containing fraction (adipo-fibroblast). Scale bars in panels A-C = 200 µm. *n* = *3–4 donors*.

Although there is an overall progression of medullar adipocyte differentiation and maturity along the 24 day time course as measured by morphology, cell lipid content and expression of lineage-stage adipogenic markers, the population of adipogenic cells is heterogeneous in all of the metrics at each time (**Fig. 1**). For example, on day 12 when many maturing lipid laden adipocytes were observed, a subpopulation of cells devoid of lipid vacuoles was also observed. To further characterize these two subpopulations of adipogenic cells, day 12 MSC-derived adipocytes were fractionated and enriched based on their lipid vacuole content and conditioned medium from these enriched populations were collected and analyzed for SDF-1 and M-CSF. Both SDF-1 and M-CSF were primarily present within the non-lipid-laden adipocyte fibroblast cultures, as measured by ELISA (**Fig. S2**). Immunofluorescence to detect RANKL from MSC-derived adipocytes at day 12 showed localization primarily within non lipid-laden cells (**Fig. 5A-C**), and this was maintained throughout the period of RANKL production (**Fig. 5D**). mRNA from both the lipid-laden and non-lipid-laden populations (**Fig. 5B-C**) was isolated and analyzed by quantitative RT-PCR for RANKL. RANKL expression was confirmed to be primarily present within the non-lipid-laden adipocyte fraction (**Fig. 5E**).These data indicate a difference in expression profile of osteoclastogenic mediators between the two morphologically distinct populations present in MSC-derived adipocyte cultures during mid-stage adipogenesis. In contrast OPG protein levels showed no population bias as measured by ELISA (data not shown).

### Adipogenic potential of RANKL enriched adipocyte population

We further tested the adipogenic potential of the non-lipid laden subpopulation of cells that were enriched for RANKL (**Fig. 5**), M-CSF, and SDF-1 (**Fig. S2**). MSC-derived adipocyte cultures on day 12 were sub-fractionated into non-lipid (adipo-fibroblasts) or lipid-laden populations, and the non-lipid-laden cell populations were re-induced to undergo adipogenesis. This secondary induction was performed by culturing cells in adipocyte induction medium for an additional 12 days. The secondary induced cells were compared to MSC-derived adipocytes after primary induction. RANKL expression in the adipofibroblasts was higher on a per cell basis when compared to culture matched MSCs (**Figure S3A-B**) in each experiment (approx. 50%±20% compared to 17%±5% in MSC's; n = 3) representative data are shown in **Figure S3E**. Lipogenesis was quantified on both a per cell basis and by total lipid vacuole accumulation (**Figure S3F&G**). Adipogenesis in the ‘adipofibroblast’ cultures was comparable to that of primary adipogenic induction in both total lipid accumulation and percent lipogenesis indicating that adipogenic potential is maintained even in the non-lipid-laden enriched fractions of MSC-derived adipocyte cultures.

### RANKL co-localization

**Figure 6:**
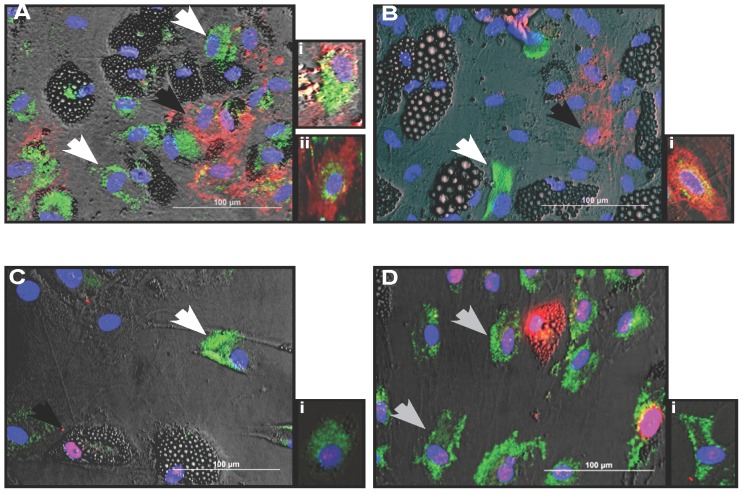
RANKL shows variant co-localization between MSC and adipocyte markers. RANKL Immunofluorescent co-staining was done to show RANKL (*green*) co-localization tendencies between the common MSC markers (**A**) CD90 (*red*) and (**B**) CD105 (*red*) and the adipocyte transcription factors (**C**) PPARγ2 (*red*) and (**D**) C/EBPα (*red*). The majority of RANKL-positive cells did not co-localize with (**A**) CD90-positive cells (**B**) CD105, or (**C**) PPARγ2 but showed some co-localization with (**D**). C/EBPα (*gray arrows*). (**Ai-Ci**) Insets shows representative instances in which there was some co-localization. (**Di**) Inset shows representative instances where co-localization was minimal.

We next tested whether the non-lipid-laden RANKL-positive cells found in MSC-derived adipocyte cultures represented undifferentiated MSC. Co-staining of RANKL and the common MSC markers CD105 and CD90 was performed (**Fig. 6A-B**). The majority of RANKL-positive cells (80%±3%; n = 3) were negative for both CD90 and CD105, while those cells that did show MSC marker co-localization with RANKL staining had a relatively lower staining intensity for either CD105 (**Fig. 6Ai**) or CD90 (**Fig. 6Bi**) and vice versa (**Figure 6Aiii**). Because of the possibility that osteoblast-like cells might be present in our cultures, which may account for the RANKL staining [46], the presence of RUNX2 in RANKL-positive cells was assessed. Although RUNX2 was present within the adipocyte cultures, the majority of RANKL positive cells were completely devoid of both RUNX2 staining (**Fig. S4**) and osterix staining (data not shown). To test whether the RANKL positive cells were committed to the adipocyte lineage, co-localization of RANKL with PPARγ and/or C/EBPα, two transcription factors important for adipocyte commitment were analyzed. Interestingly, there was little RANKL and PPARγ co-localization (**Fig. 6C**). In contrast, RANKL co-localization with C/EBPα was observed (**Fig. 6D&Di**).

### Adipofibroblast co-culture with PBMNC

**Figure 7:**
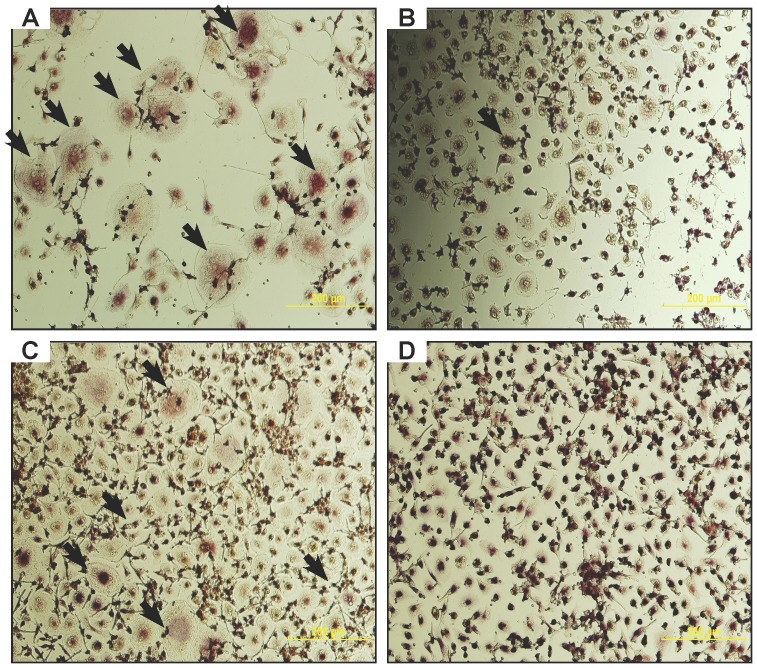
Osteoclast-like formation of adipofibroblast/PBMNC co-cultures. (**A-D**) the osteoclast regulating potential of the adipofibroblast derived from MSC-derived adipocyte cultures was assessed by TRAP staining. After 15 days of co-culture with peripheral blood mononuclear cells (PBMNC) and was compared to (**C**) positive controls where PBMNCs cultures were given 50 ng/ml of M-CSF and 60 ng/ml RANKL in DMEM:RPMI (1/1) medium and (**D**) untreated controls where PBMNC were cultured in the same mixed basal medium only. (**C**) Multinucleated cells formed after treatment with M-CSF and RANKL for 15 days showing a distinct osteoclast like cell phenotype including multi-nucleation, TRAP positivity and a large diameter. Positive osteoclasts-like cells were numerous in (**A**) co-cultures with adipofibroblasts and (**C**) positive controls (*black arrows*) while scarce in (**D**) co-cultures with dermal fibroblasts. (**A**) Multi-nucleated TRAP positive osteoclast-like cells formed in ‘adipofibroblast’ PBMNC co-cultures after 15 days in culture. Osteoclast-like cell formation was comparable to that of the (**B**) positive controls while no significant formation of multi-nucleated TRAP-positive osteoclast-like cell formation was seen in (**B**) dermal fibroblast and PBMNC co-cultures. Scale bar in panel A = 200 µm for panels A-B and 300 µm for panels C-D. *n* = *3–4 donors for both MSC and PBMNC donors respectively*.

Co-cultures of adipofibroblasts and peripheral blood mononuclear cells (PBMNCs) were used to show the capability of ‘adipofibroblasts’ to support formation of TRAP-positive multi-nucleated osteoclast-like cells. When compared with positive controls of PBMNC supplemented with recombinant RANKL and M-CSF, co-cultures with adipofibroblasts exhibited comparable levels of TRAP-positive staining and multinucleated osteoclast-like cell formation (**Fig. 7A&C**). Dermal fibroblasts serving as a negative control did not show comparable levels of multi-nucleation and TRAP positivity in co-cultures with PBMNCs (**Fig. 7B&D**).

## Discussion

The classical view of bone remodeling involves the coupling relationship between the osteoblast and osteoclast, in which early osteoblasts express RANKL to support osteoclast formation, and late osteoblasts express OPG while reducing expression of RANKL to inhibit osteoclast formation. We and others [47]–[50] provide evidence that this may not be exclusive to the osteoblast lineage and may be shared by other early stage cell phenotypes of the MSC lineage. This suggestion is supported by the observations that osteoclast number is minimally affected, and in some cases increased, in increased marrow adiposity [18], [21] even in the presence of decreased osteoblast activity [18]. In this study, our aim was to determine whether or not cells of the medullary adipocyte lineage have the potential to regulate osteoclast formation through the expression of osteoclastogenic regulatory molecules. We hypothesized that cells of the medullary adipocyte lineage influence osteoclastogenesis through the expression of positive and/or negative osteoclast mediators based on their developmental stage, similar to the expression of osteoclastogenic mediators by cells along the osteogenic lineage [6], [7].

Using a well-characterized model of medullary adipocyte differentiation from marrow-derived MSCs, mRNA and protein expression of several important osteoclast mediators was measured in adipocyte cells at various lineage stages along their differentiation from MSCs. [28]. We report here novel findings of mRNA and protein expressed in developmentally regulated patterns for RANKL, OPG, and M-CSF in adipocyte lineage cells. In the case of RANKL, the majority of expression was observed on a previously uncharacterized cell lineage stage of medullary adipocytes. These RANKL positive cells appear to be early-stage, pre-lipid-laden adipogenic cells that also express C/EBPα, but not PPARγ2 or phenotypic markers characteristic of early osteoblasts or MSCs.

Studies looking at the potential relationship between marrow stromal derived adipocytes and osteoclasts have been conducted in both mice and humans [19], [24], [32], [49]. In humans, isolated primary medullary adipocytes were shown to express M-CSF, OPG, and, with the addition of dexamethasone, RANKL [51], [52]. Unfortunately, a major drawback of ex vivo isolation of medullary adipocytes is the heterogeneous mixture of cell types present before and after culture [53], thereby preventing reliable tissue characterization due to the possible inclusion of a number of stromal cell phenotypes. Similarly, RANKL expression was observed in mouse medullary adipocytes [19], [20]. Although RANKL expression was detected in these medullary adipocyte cultures, attempts to further characterize the specific cell phenotype responsible for the RANKL expression was not made. Collectively, these studies were able to identify a capacity of early adipocyte cultures to support osteoclast formation [20], [24] and express RANKL [19], [20], [24].

Consistent with these previous studies a possible direct relationship between adipocytes and osteoclasts through the expression of osteoclast mediating factors M-CSF and RANKL is also suggested by our data. Further, we show that MSC-derived adipogenic cells can support osteoclast-like cell development in co-cultures with PBMNCs. In addition to this, our data show that the majority of RANKL expression and osteoclast support potential by cells in our MSC-derived adipocyte culture system is by non-lipid-laden adipocyte-like cells termed here as ‘adipofibroblasts’. Adipose tissue is a highly heterogeneous tissue and contains a diverse population of many different cell phenotypes [11], [54]–[57]. Included within this heterogeneous population of cells are MSC-like cells [55], [58], committed pre-adipocytes [11], [57], [59]–[63], and uncommitted progenitor pools [56]. To determine which of these non-lipid-laden cell types contribute to the RANKL expression and osteogenic support potential observed in our MSC-derived medullary adipocytes cultures, a series of co-localization studies were conducted using antibodies to RANKL and phenotypic markers that identify MSCs, osteoblast lineage cells, and cells of the adipogenic lineage. Markers for MSC phenotype, CD105 and CD90, although present, were shown not to co-localize with the RANKL expressing cells. CD105 and CD90 have been shown previously to be downregulated during MSC adipogenic and osteogenic differentiation in cultured umbilical cord MSCs [64]. However, studies focusing on individual cell identity during adipocyte differentiation were able to show that there does exist small populations of pre-adipocytes both in vitro and in vivo that are CD105 and/or CD90 positive [57], [65], [66]. The results presented in this study thus indicate that the RANKL positive populations in these MSC-derived adipocyte populations were neither uncommitted MSCs nor CD90-/CD105-positive pre-adipocytes. Furthermore, there was no evidence of these RANKL-expressing cells having an early osteoblastic lineage phenotype as indicated by the absence of Runx2 expression. Interestingly, the majority of RANKL-positive cells were capable of expressing C/EBPα but did not show significant co-localization with PPARγ. Collectively, these data show that the majority of RANKL-expressing cells observed in the MSC-derived medullary adipocyte cultures are from non-lipid-laden fibroblast-like cells that appear to be very early stage medullary adipocyte lineage cells based on their positive expression of C/EBPα and lack of PPARγ2 expression. Our findings are in contrast to data from studies involving mouse medullary adipocytes, in which cells that were capable of expressing RANKL and supporting osteoclast-like cell formation were PPARγ positive [19], [20], but are similar to another study where RANKL expression was linked to C/EBPβ and C/EBPδ but not PPARγ [50]. Moreover, there have been no studies that have identified the presence of any PPARγ response elements (PPRE) or PPARγ/RXR binding motifs [50], [67], [68] in the promoter region of the RANKL gene suggesting that PPARγ has neither an inductive or inhibitive property on RANKL gene expression. In future studies it would be of use to further identify whether or not there are any pro-adipogenic candidate transcription factors that are capable of binding to either the RANKL or OPG promoter regions.

RANKL/C/EBPα -positive adipofibroblast cells are first observed around day 6 (data not shown) of the 25 day time course of induction and differentiation of MSCs into medullary adipocytes; however, these cells persist throughout the time course as other cells within the cultures become lipid-laden and express markers characteristic of maturing adipocytes. However, when isolated and reintroduced into culture under adipogenic induction conditions, the majority of these adipofibroblasts differentiate into mature, lipid-laden adipocytes; while a subpopulation of the adipofibroblast remain– as observed in the original cultures. These observations suggest the presence of a mechanism within the microenvironment of our MSC-derived medullary adipocyte cultures that acts to prevent the developmental maturation of these early stage adipofibroblasts.

There are several mechanisms that have been associated with the maintenance of cells in an intermediate pre-adipogenic state [53], [69]–[72]. For instance, pre-adipocyte factor 1 (pref-1), expressed by early adipocytes, has been shown to inhibit progression down the adipogenic lineage in a paracrine/juxtacrine manner by, among other mechanisms, inhibiting PPARγ expression and activity [73]–[75]. With respect to these data, two important findings we show here should be considered: that the ‘adipofibroblasts’ present within our cultures maintain an adipogenic potential comparable to primary induced adipocyte cultures; and that these cells are largely PPARγ negative while capable of expressing other related early pro-adipogenic factors (data not shown), including C/EBPα. These results reinforce the possibility that an inhibitory mechanism is in place (possibly mediated by pref-1 and/or other factors) that controls adipogenic commitment as defined by PPARγ expression and thereby inhibits adipogenesis progression in these MSC-derived adipocyte cultures. Interestingly, in a recent mouse study, a linkbetween pref-1 expressing cells in the marrow and osteoclast activity was observed. The study, by Takeshita et al., showed that ‘intermediate’ pref-1 expressing adipocytes were positive for RANKL while capable of osteoclast support after ex vivo isolation and co-culture with bone marrow macrophages. Furthermore, the presence of these cells increase with mouse age. This study provides clear evidence of the existence of a RANKL expressing ‘intermediate’-like adipocyte in mouse marrow and the possibility that this might be inductive to the activity of osteoclasts. Further experiments are needed to identify the mechanism by which these cells are maintained in this proposed intermediate pre-adipocyte-like state and whether this state intensifies the RANKL-positive phenotype and osteoclast activity in the marrow. In addition to pref-1, both canonical and non-canonical Wnt signaling has been identified as a candidate control of adipogenesis [70], [76]. Wnt10b and its receptor Lrp5 have been implicated in the maintenance of adipose tissue by inhibiting adipogenesis and maintaining a precursor pool; this is known as pro-adipogenic Wnt signaling [77].

In summary, RANKL is expressed in a distinct subpopulation of MSC-derived medullary adipocyte lineage cells, and these, previously uncharacterized cells, are capable of supporting osteoclast-like cell formation in co-cultures. We, along with another recent study done in mice [50], show that there is a link between early stage adipocytes and the expression of RANKL. These data confirm earlier studies that suggest a role for human medullary adipose tissue in the regulation of osteoclastogenesis and identifies a possible cellular target to further characterize with hope of better understanding the proposed osteoclast support potential of medullary adipocytes in vivo.

## Supporting Information

Figure S1 **High Confluence MSCs show altered phenotype.** To assess change in MSC phenotype after extended time in culture, MSCs in Growth Medium were cultured at 200% confluence and total RNA was isolated at day 0 and day 21 after plating and analyzed **(A)** RUNX2; **(B)** COL1; **(C)** Osteocalcin; and **(D)** RANKL using qPCR.
(TIFF)

Figure S2 **M-CSF and SDF-1 expression in enriched adipo-fibroblasts.** Three day conditioned medium collected from day 12 MSCs (*black bars*), day 12 adipocytes (*light gray bars*), adipo-fibroblasts (*dark gray bars*), or the lipid-laden fraction (*white bars*) at the indicated timepoints was analyzed using ELISA for **(A)** M-CSF and **(B)** SDF-1. M-CSF expression was analyzed at time of split and subsequent days after enrichment and plating whose methods are described in the Materials & Methods section. **(B)** SDF-1 expression in adipo-fibroblasts was analyzed six days after enrichment and was compared to the lipid-laden fraction.
(TIFF)

Figure S3 **RANKL-positive enriched subpopulation is capable of adipogenic induction.** MSC-derived adipocyte cultures were allowed to undergo adipogenesis for 12 days. **(F)** graphical representation of RANKL expression which was observed, by **(A,B)** immunofluorescence, to be higher in the enriched non-lipid-laden sub-populations (MSCs-*dark bar*; adipofibroblasts- *white bar*). The potential for adipogenesis in the enriched non-lipid-laden **(B,D)** RANKL-positive subpopulation of these 12-day cultures was compared to that of **(A,C)** culture matched MSC controls after both populations were re-induced for adipogenesis 24 hours after enrichment. **(F)** Percent lipid-laden cells was quantified by counting Oil Red O + stained cells and of several fields resulting in a percentage of average of lipid-containing cells vs.average total cell count. Both lipid-laden and non-lipid-laden populations showed an ability to form adipocytes as observed by **(C,D)** Oil Red O staining in both. **(E)** Total lipid accumulation was measured by counting Oil Red O-positive cells and was shown to be comparable in culture matched MSCs and parent MSC-derived adipocyte cultures.
(TIFF)

Figure S4 **Runx2 expression in RANKL positive MSC-derived adipocytes.** Co-staining of RANKL (*green*) and RUNX2 (*red*) was done in order to show potential co-localization between the two molecules. **(A)** In the Osteosarcoma (SaOs-2) positive controls, co-localization (*yellow arrow*) was expected in the Osteosarcoma. **(B)** In MSC-derived adipocyte cultures at day 12 highly RANKL-positive cells (*white arrow*) showed no evidence of RUNX2 expression. In cells that were positive for RUNX and RANKL showed RUNX2 expression mainly in the cytoplasm of cells (*yellow arrows*).
(TIFF)

Table S1 **List of gene primers used for quantitative PCR analysis.**
(DOCX)
