# Supplementary figures and images for: Identification of a Subpopulation of Marrow MSC-Derived Medullary Adipocytes That Express Osteoclast-Regulating Molecules: Marrow Adipocytes Express Osteoclast Mediators
Source: PLoS One. 2014 Oct 10;9(10):e108920. doi: 10.1371/journal.pone.0108920 (PMC4193782; doi:10.1371/journal.pone.0108920)

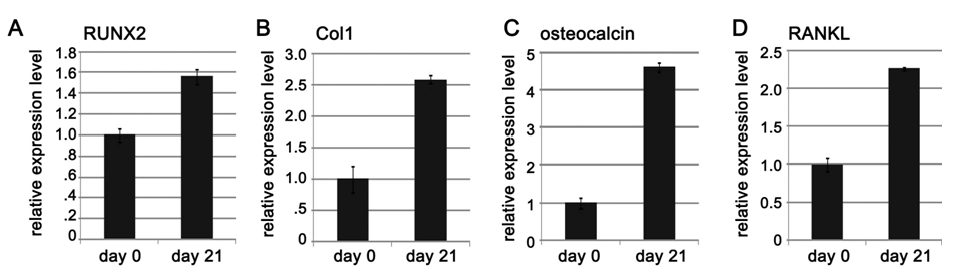

Supplement: Figure S1 — High Confluence MSCs show altered phenotype. To assess change in MSC phenotype after extended time in culture, MSCs in Growth Medium were cultured at 200% confluence and total RNA was isolated at day 0 and day 21 after plating and analyzed (A) RUNX2; (B) COL1; (C) Osteocalcin; and (D) RANKL using qPCR. (TIFF) [file pone.0108920.s001.tiff]

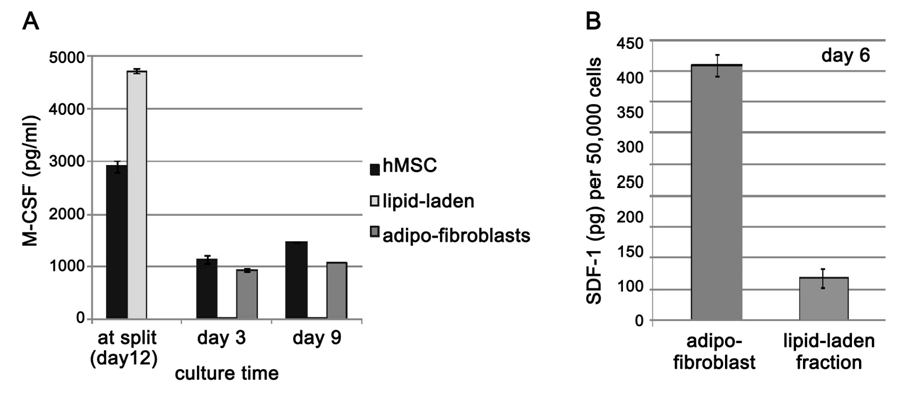

Supplement: Figure S2 — M-CSF and SDF-1 expression in enriched adipo-fibroblasts. Three day conditioned medium collected from day 12 MSCs (black bars), day 12 adipocytes (light gray bars), adipo-fibroblasts (dark gray bars), or the lipid-laden fraction (white bars) at the indicated timepoints was analyzed using ELISA for (A) M-CSF and (B) SDF-1. M-CSF expression was analyzed at time of split and subsequent days after enrichment and plating whose methods are described in the Materials & Methods section. (B) SDF-1 expression in adipo-fibroblasts was analyzed six days after enrichment and was compared to the lipid-laden fraction. (TIFF) [file pone.0108920.s002.tiff]

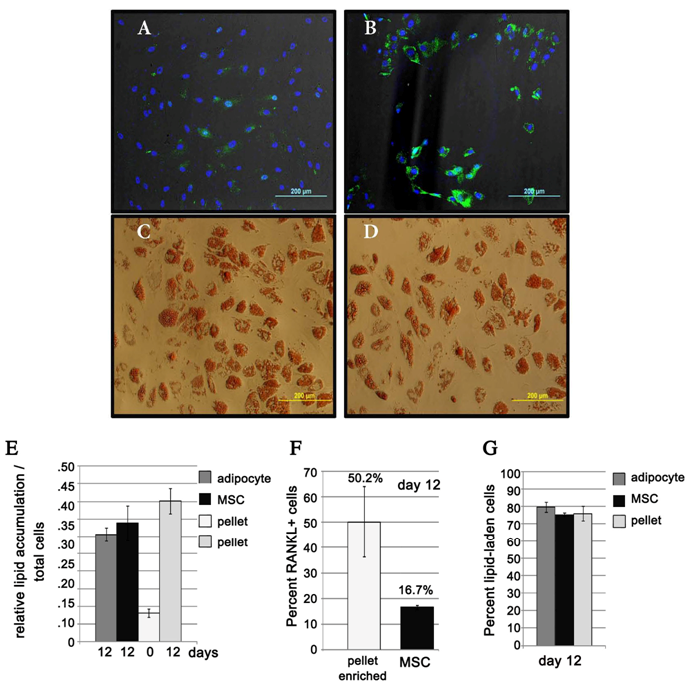

Supplement: Figure S3 — RANKL-positive enriched subpopulation is capable of adipogenic induction. MSC-derived adipocyte cultures were allowed to undergo adipogenesis for 12 days. (F) graphical representation of RANKL expression which was observed, by (A,B) immunofluorescence, to be higher in the enriched non-lipid-laden sub-populations (MSCs-dark bar; adipofibroblasts- white bar). The potential for adipogenesis in the enriched non-lipid-laden (B,D) RANKL-positive subpopulation of these 12-day cultures was compared to that of (A,C) culture matched MSC controls after both populations were re-induced for adipogenesis 24 hours after enrichment. (F) Percent lipid-laden cells was quantified by counting Oil Red O + stained cells and of several fields resulting in a percentage of average of lipid-containing cells vs.average total cell count. Both lipid-laden and non-lipid-laden populations showed an ability to form adipocytes as observed by (C,D) Oil Red O staining in both. (E) Total lipid accumulation was measured by counting Oil Red O-positive cells and was shown to be comparable in culture matched MSCs and parent MSC-derived adipocyte cultures. (TIFF) [file pone.0108920.s003.tiff]

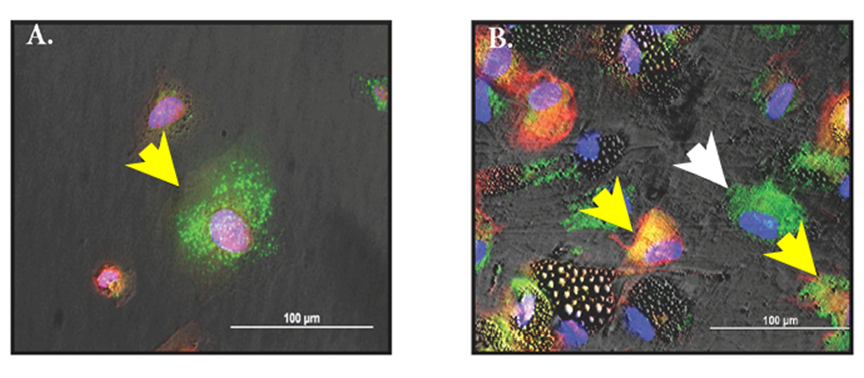

Supplement: Figure S4 — Runx2 expression in RANKL positive MSC-derived adipocytes. Co-staining of RANKL (green) and RUNX2 (red) was done in order to show potential co-localization between the two molecules. (A) In the Osteosarcoma (SaOs-2) positive controls, co-localization (yellow arrow) was expected in the Osteosarcoma. (B) In MSC-derived adipocyte cultures at day 12 highly RANKL-positive cells (white arrow) showed no evidence of RUNX2 expression. In cells that were positive for RUNX and RANKL showed RUNX2 expression mainly in the cytoplasm of cells (yellow arrows). (TIFF) [file pone.0108920.s004.tiff]
