# Supplementary material for: Identification of a Subpopulation of Marrow MSC-Derived Medullary Adipocytes That Express Osteoclast-Regulating Molecules: Marrow Adipocytes Express Osteoclast Mediators
Source: PLoS One. 2014 Oct 10;9(10):e108920. doi: 10.1371/journal.pone.0108920 (PMC4193782; doi:10.1371/journal.pone.0108920)
Supplement: Table S1 — List of gene primers used for quantitative PCR analysis. (DOCX) [file pone.0108920.s005.docx]

| **GENE** | **Forward Sequence** | **Reverse Sequence** |
| --- | --- | --- |
| PPARγ2 | ATTGACCCAGAAAGCGATTC | CAAAGGAGTGGGAGTGGTCT |
| C/EBPα | GCAAACTCACCGCTCCAATG | TTAGGTTCCAAGCCCCAAGTC |
| Adiponectin | TCTGATTCCATACCAGAGGGGCTCA | CAGGACTCCGGGCCCTTGAGT |
| Leptin | GAACCCTGTGCGGATTCTTGTG | CGTTTCTGGAAGGCATACTGGTGAG |
| M-CSF | CAGGCCCCTGCCCCGTTTTAA | ACGGAGAGACTGTTCTGTGCGTCCA |
| RANKL | CAGCACATCAGAGCAGAGAAAGC | CCCCAAAGTATGTTGCATCCTG |
| OPG | AGCTTGCACCACTCCAAATCC | GGGGACCACAATGAACAACTTG |
| SDF-1 | CCGCTAGACCCACTCGAGGAAAA | TGGCAAACCTCAGGCCCGAT |
| Beta-actin | TGTGCCCATCTACGAGGGGTATGC | GGTACATGGTGGTGCCGCCAGACA |
